# Design an efficient data driven decision support system to predict flooding by analysing heterogeneous and multiple data sources using Data Lake

**DOI:** 10.1016/j.mex.2023.102262

**Published:** 2023-06-22

**Authors:** Sreepathy H V, B Dinesh Rao, Mohan Kumar J, B Deepak Rao

**Affiliations:** Manipal School of Information Sciences, MAHE, Manipal, 576104, India

**Keywords:** Flood Prediction, Data Lake, Flood causing vital factors, Inferential Statistics

## Abstract

Floods are the most common natural disaster in several countries throughout the world. Flooding has a major impact on people's lives and livelihoods. The impact of flood disasters on human lives can be mitigated by developing effective flood forecasting and prediction models. The majority of flood prediction models do not take all flood-causing factors into account when they are designed. It is difficult to collect and handle some of these flood-causing variables since they are heterogeneous in nature. This paper presents a new big data architecture called Data Lake, which can ingest and store all important flood-causing heterogeneous data sources in their raw format for machine learning model creation. The statistical relevance of important flood producing factors on flood prediction outcome is determined utilizing inferential statistical approaches. The outcome of this research is to create flood warning systems that can alert the public and government officials so that they can make decisions in the event of a severe flood, reducing socioeconomic loss.

• Flood causing factors are from heterogeneous sources, so there is no big data architecture for handling variety of data sources.
• To provide data architectural solution using data lake for collecting and analysing heterogeneous flood causing factors.
• Uses inferential statistical approach to determine importance of different flood causing factors in design of efficient flood prediction models.

## Graphical abstract

**Figure:**
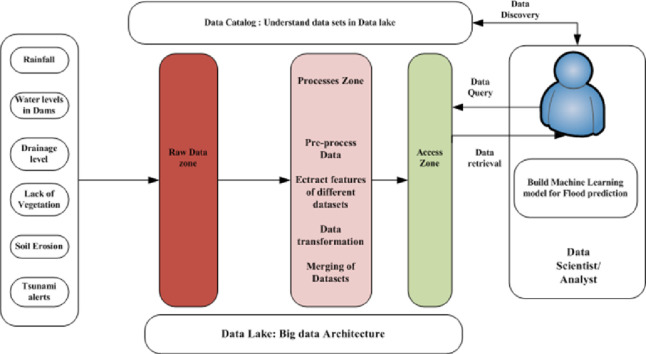

Specifications table

**Table:** 

Subject area:	Computer Science
More specific subject area:	Big Data Architecture
Name of your method:	Inferential Statistics
Name and reference of original method:	NA
Resource availability:	NA

## Introduction

Floods are produced by rain, lack of vegetation, damaged dams, soil erosion, inadequate drainage, storm surges and tsunamis, rivers filled with silt, melting snow and ice, etc. Indian monsoon, the world's most prominent monsoon system, affects India and its waterways. In July and August 2019, persistent rainfall caused flooding in 13 Indian states. A million people were displaced and 200 died. Most hit were Karnataka and Maharashtra. Heaviest monsoon in 25 years. In June-October 2019, almost 1600 individuals died [1]. Heavy rains in July–September 2019 flooded 13 Indian states [2]. 500 lost, 1000 killed, and many homes destroyed, according to reports. Floods destroy lives, infrastructure, society, and state economy. Floods are natural tragedies produced by high rainfall or other hazardous situations. Hydrological, meteorological, geo-graphical, topographical, and planning issues can cause floods [3]. Heavy rainfall and water discharge are the most typical causes of floods. Storm and cycle changes are metrological conditions [4]. Geographic conditions cause state-to-state water flow, and planning issues include river siltation, insufficient drainage systems, etc. Floods influence many lives and society. Flood types include Coastal flood (Surge), River flood (Fluvial) and Surface flood (Pluvial) [5]. Early flood prediction and detection saves lives and livelihoods. Many flood prediction models simply include rainfall and river water levels, however including the later mentioned all flood causing factors in the dataset might improve data analysis/machine learning models' classification performance. Diverse flood-causing variables necessitate flexible big data architecture that can manage data from many types and heterogeneous sources. For higher performance and accuracy, flood detection models must extract features from diverse data. Web-based dashboards or Flood alerting systems with SMS/phone calls or Flood warnings systems must be combined to alert the public and government authorities so that required safeguards can be taken, sparing many people's lives.

## Literature review

The following is a list of possible solutions for collecting, analyzing, and predicting flood data. Monitoring and predicting floods can be done by using sensors such water level, water flow, ultrasonic and magnetic field sensors [6]. Wi-Fi connectivity is used to transfer sensor data to Cloud-based IoT platforms via the Internet of Things [7]. When it comes to retrieving time series data, WSN is an information retrieval component that yields the best results possible [8]. Fog servers can be used to analyze data from mobile edge nodes, which then pass it on to the sensing layer, which contains a variety of static and mobile sensors IoS nodes [9]. A similar approach combining IIoT and fog is also used to sample flood prevention and causation properties [10]. A geographic information system (GIS) is a conceptual framework for collecting and analyzing geographical and geographic data [11]. Flood detection and early warning can be made more cost-effective by utilizing satellite-based rainfall and geospatial datasets, especially in areas where local data is scarce or non-existent [12].Flood prediction requires greater data analysis. Big data analytics is a developing technology that helps companies make business decisions. An algorithm based on the advanced object detection CNN models (YOLOv3 and YOLOv4) for the detection of forest smoke aims to reduce losses of billion [13]. ANN-based flood prediction aims to improve system scalability and dependability [7]. Deep learning algorithm Multilayer Perceptron can anticipate flood events based on rainfall time series data and water levels. Multilayer Perceptron had a Mean Absolute Percentage Error of 3.64 percent, meaning the system created 3.64 percent more error than the genuine number used for testing. Multilayer Perceptron predicts canal water level better than multiple regression linear models [8]. Layered techniques comprising of both regional fog server and cloud permit training, testing, analysis, and decision making that values flood probability on an ordinal scale. The OFFM-ANFIS features seven modified ANFIS models that anticipate floods using training data and sensory data. The flow of raw and analysed data from OFFM-ANFIS is tiered so that more influential parameters have a large impact [9]. SVD-based characteristics reduction strategy for K-mean clustering algorithm is used to estimate the current condition of flood and flood rating in any place [10]. Convolutional Neural Networks that extract visual features and bidirectional Long Short-Term Memory networks that extract semantic information from textual metadata may predict flood using picture datasets [14]. Multi-parameter analysis Multi-criteria evaluation approaches like Boolean and Weighted Linear Combination can be used to calculate the weights of each component in predicting the flood [11]. Flood prediction models must be validated by Benchmark hydrological models.

Hybrid Machine Learning models are employed to improve the performance and efficiency of flood prediction models like Bidirectional Gated Recurrent Unit (BiGRU) multi-step [15], LSTM–GRU-based model [16] and adaptive neuro-fuzzy inference system (ANFIS) combined with ant colony optimization (ACO) algorithm which optimize model parameters for predicting flood accurately [17].

Early prediction technology sends SMS and social media alerts to prevent material and human loss. Government and public can respond effectively to looming risk with this knowledge [6]. Microcontroller based systems integrated with GSM Modules are used to alert the public using SMS and android applications [18]. Recent web technology improvements allow organizations and institutions to bring desktop-level data analysis and visualization to web-based systems. Web systems are frequently employed in flood planning, preparedness, response, and recovery [19].

A flood prediction system needs flood-causing parameter data. Data analyst's challenge is to obtain all impacting factors. Data varieties, sources, and silos make data acquisition difficult. Data lake was designed to handle massive data challenges, particularly variety issues. A data lake is a massive data storage, management, and analysis facility. Dixon suggested data lakes as a solution to data marts and data warehouses, which can only address a fraction of business queries. Dixon views the lake a huge repository for raw data from various sources, allowing users to explore, collect, and analyze information [20,21]. Hadoop-based Big Data Lake ecosystem can be used to store smart grid digital data, photos, and videos for unsupervised data mining and visualization [22]. Novel data lake architecture improves data analytics by reducing data ingestion time. It removes data silos and improves analytics by permitting third-party data connectivity [23].

This paper is structured as follows: section 3, illustrates rationale of research. Section 4, presents system design. Section 5, describes about data collection and data sources. Section 6, present approach to prove significance of data collected statistically using inferential data analysis. Section 6, highlights implication of the study. Section 7, draw conclusion of proposed work.

## Rationale of the research

1. Floods has devastating impact on individuals and communities; which in-turn has social, economic, and environmental consequences. Flood causes loss of human lives, livestock, livelihood and property.
2. Multiple Flood causing factors which has significant influence are identified and features of these variables are extracted, mixed data model is designed and evaluated to predict future trends of flood occurrences.
3. As traditional database architecture fails to handle variety of data, Scalable and agile Data lake architecture enables to design and build an efficient, cost effective, pre-emptive flood prediction and warning/alerting system.
4. Positivist paradigm using Quantitative data analysis approach is applied for design of Efficient Flood prediction and warning system.

## System design

This work attempts to obtain extensive and accurate entities that causes the flood and designing a system to which predicts the occurrence of the flood and which then alerts the population in large to take necessary precautions thus this work falls under Diagnostic study based on the intent of the research. This study focusses on analyzing historical and real time data which involves Quantitate data exploration, hence this research can also be categorized under Evaluation study. Since this work involves analysis of Quantitative data, Analytical study of Research methods are employed.

Proposed work collects flood-related data variables from Data Lake's raw data zone. This project employs Inferential Quantitative research to construct machine learning models to predict/infer flood events and alert authorities.

**Fig. 1:**
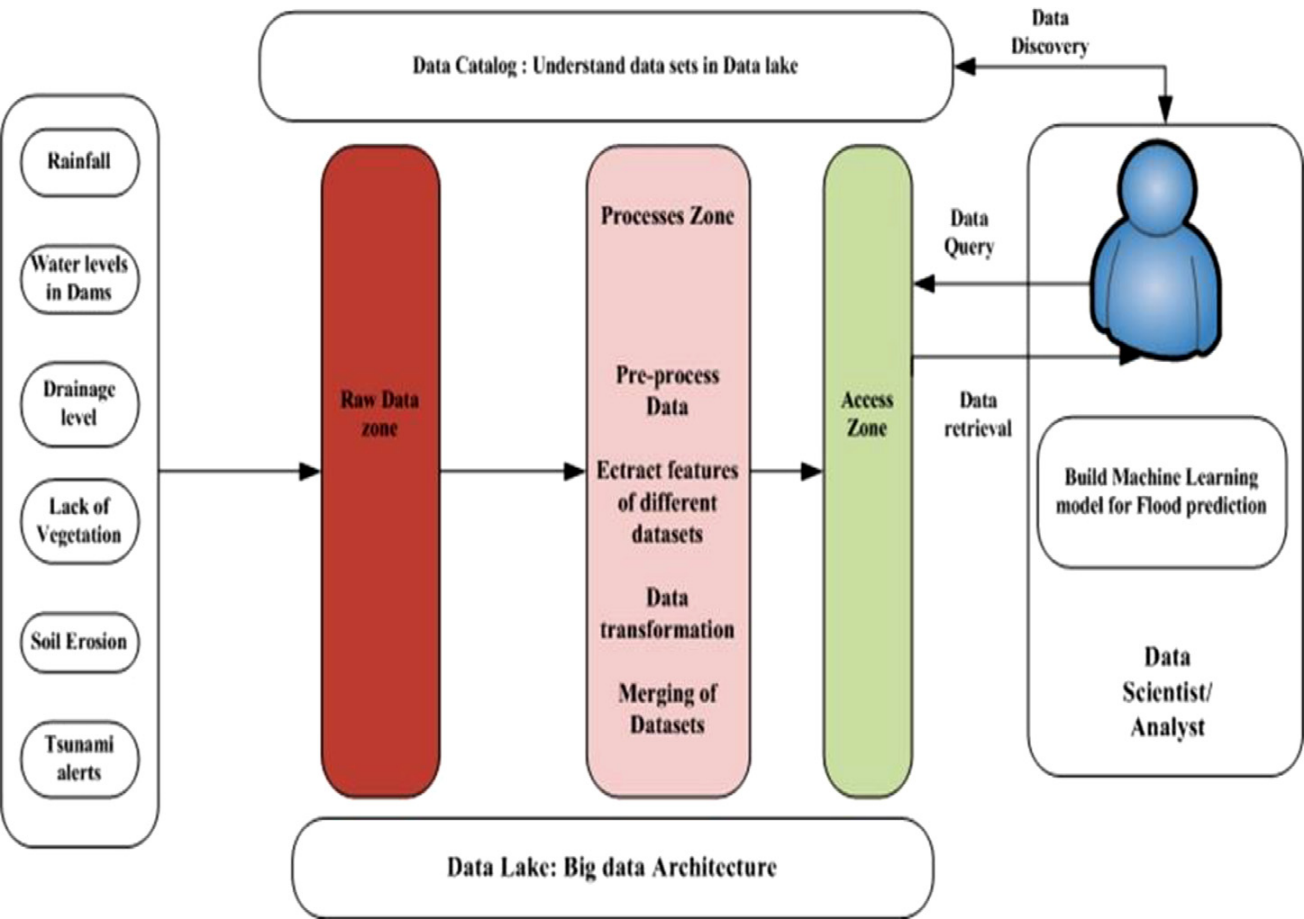
Overview of system diagram of data lake based decision support system for flood prediction model.

Data Lake handles heterogeneous data sources. To find related objects in Data Lake, Web-based Catalog are used. Data scientists can find more meaningful information from various datasets with the same context, i.e. flood-causing features, which improves model classification. Fig. 1 provides an overview of system diagram of Data lake based decision support system for Flood prediction model.

### Design a data lake architecture for ingestion and processing of vital flood causing data attributes

**Fig. 2:**
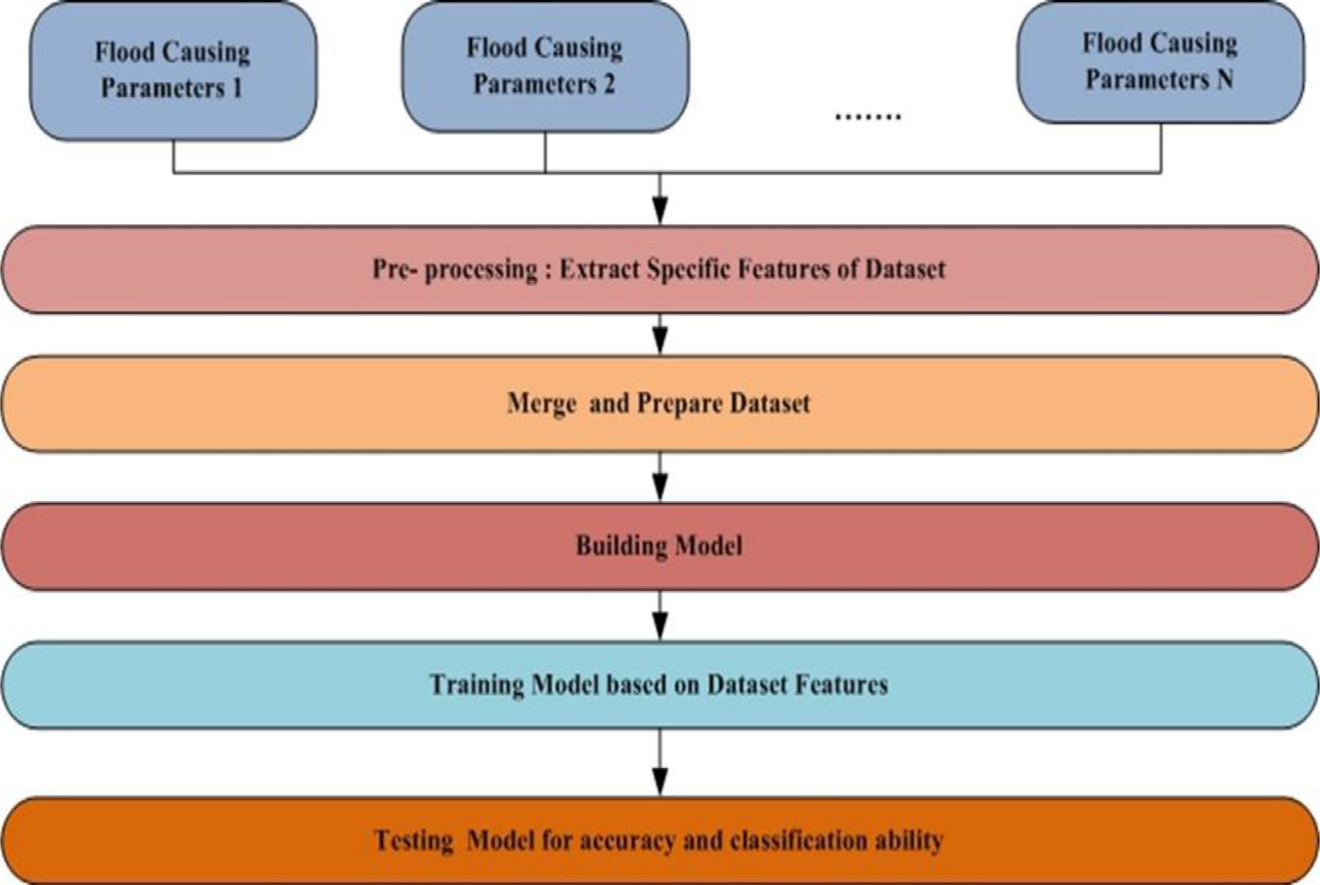
Flow diagram of designing flood detection machine learning model using data lake.

Data Lake architecture reduces data-silos so users can easily ingest heterogeneous data. Rainfall, lack of vegetation, damaged dams, soil erosion, poor drainage, storm surges and tsunamis, silted rivers, melting snow and ice, etc. produce floods. Multiple-data-type machine learning models improve classification. Traditional big data architecture can't process, scale, and be agile with semi-structured and unstructured data. Data Lake architecture can overcome these flaws and store raw data from numerous sources. Data analyst can query and retrieve multiple data kinds from Data Lake's access zone. Data analyst can create a machine learning model to predict flood by training prepared data set and testing model performance. Flow Diagram. Fig. 2 provides flow of designing a flood detection machine learning model.

### Develop a flood warning or alert system using web based dashboard to visualize flood impact on particular geographical region on globe

**Fig. 3:**
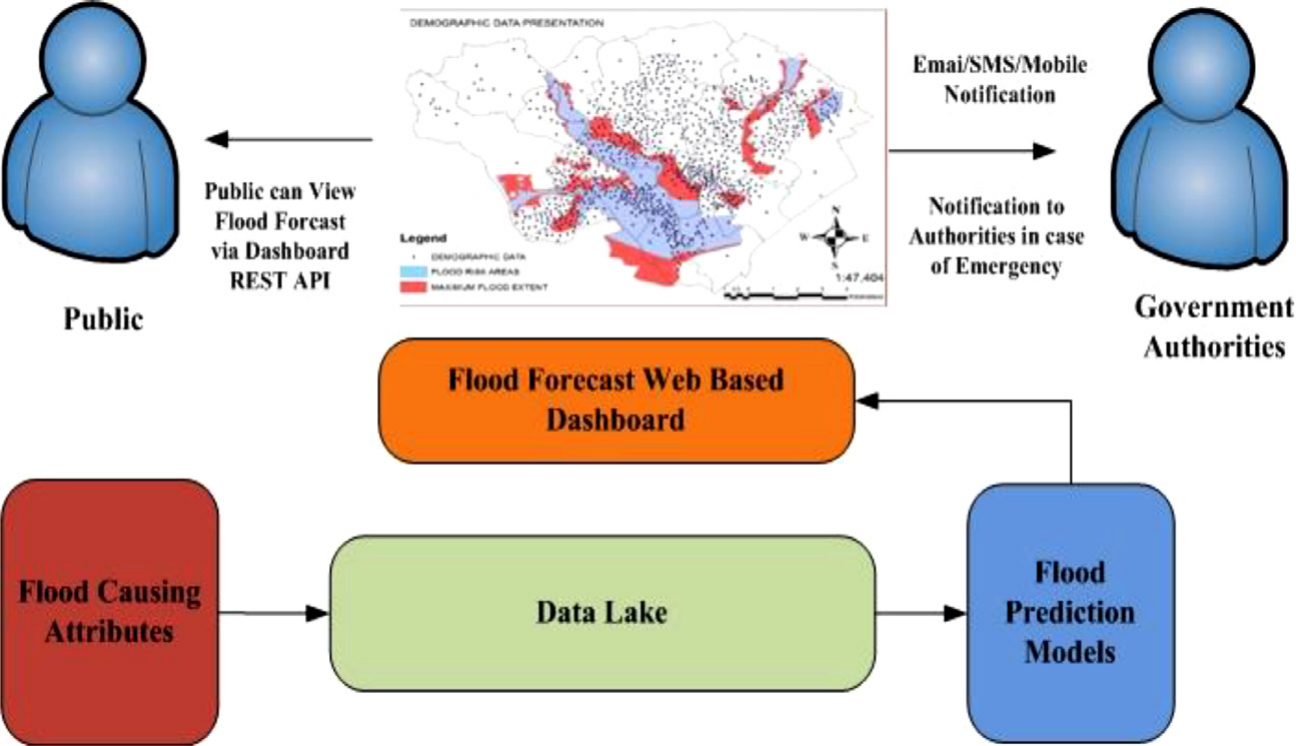
Overview of flood warning system based on data lake using web dashboard.

Flood warning system need to send appropriate forecasts to authorities or communities of those potentially affected by the developing flood, as soon as possible. Instant access to project data is available 24/7 through a cloud-based data center. Monitoring data can be viewed in real time or as a graph to identify trends. Real-time automated alerts can be sent via text or email when specified parameters exceed predefined limits. Cloud based Data Lake lays a foundation to create web dashboard to visualize real time flood forecasts and can be integrated with cloud notification services which can alert concerned authorities via Email/SMS/Mobile Notification to take necessary precautions. Cloud based Flood forecast dashboard can be integrated via REST API calls to other Web applications also get alerts and notification. Fig. 3 provides an overview of Flood warning system based on Data Lake using Web Dashboard.

### System design architecture for data preparation and model building

Data related to flood causing attributes are ingested in to Data Lake Raw data zone to store data in its raw format. Rainfall and Reservoir water level data is a structured data stored in CSV format, Sewage/Underground drainage data is a secondary source data captured via Embedded systems is a real time stream data ingested in to data lake using real time stream processing services, Land mass cover and Tsunami alerts data are acquired using satellite images and they are an unstructured data. Data lake manages and categories data based on metadata. Business and Technical metadata of these flood related data are extracted and indexed in to search engine so Data analyst can discover Flood related data via Data Catalog.

**Fig. 4:**
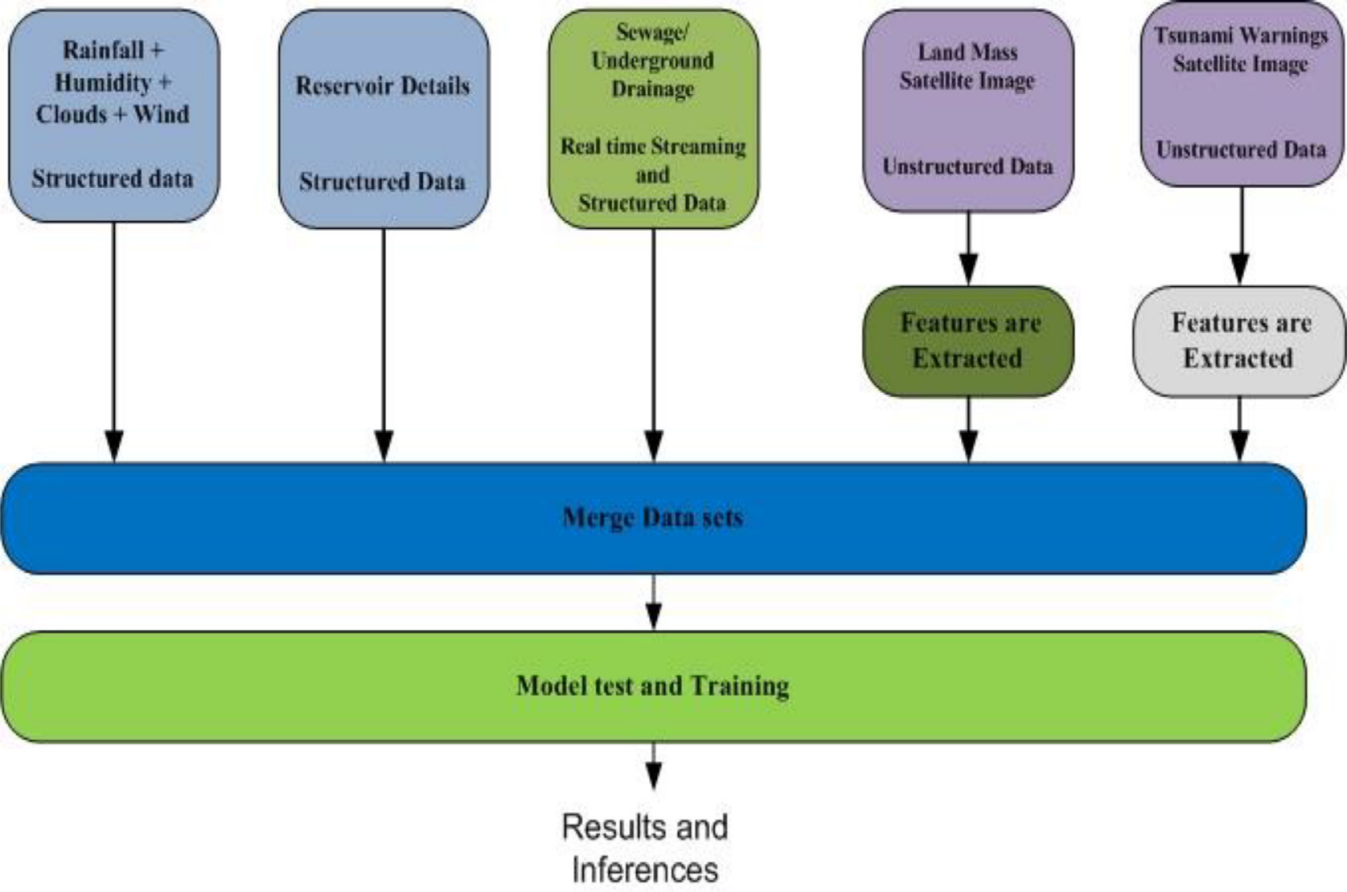
System design for flood prediction model.

Dataset preparation for Flood prediction model involves extraction of specific fea-tures from Unstructured images and merging these features with structured data and real time stream data. Fig. 4 provides an overview of System Design Architecture for Flood Prediction Model. Data prepared is used for training model and model can be evaluated using testing dataset.

### Flood prediction model deployment for alert management

**Fig. 5:**
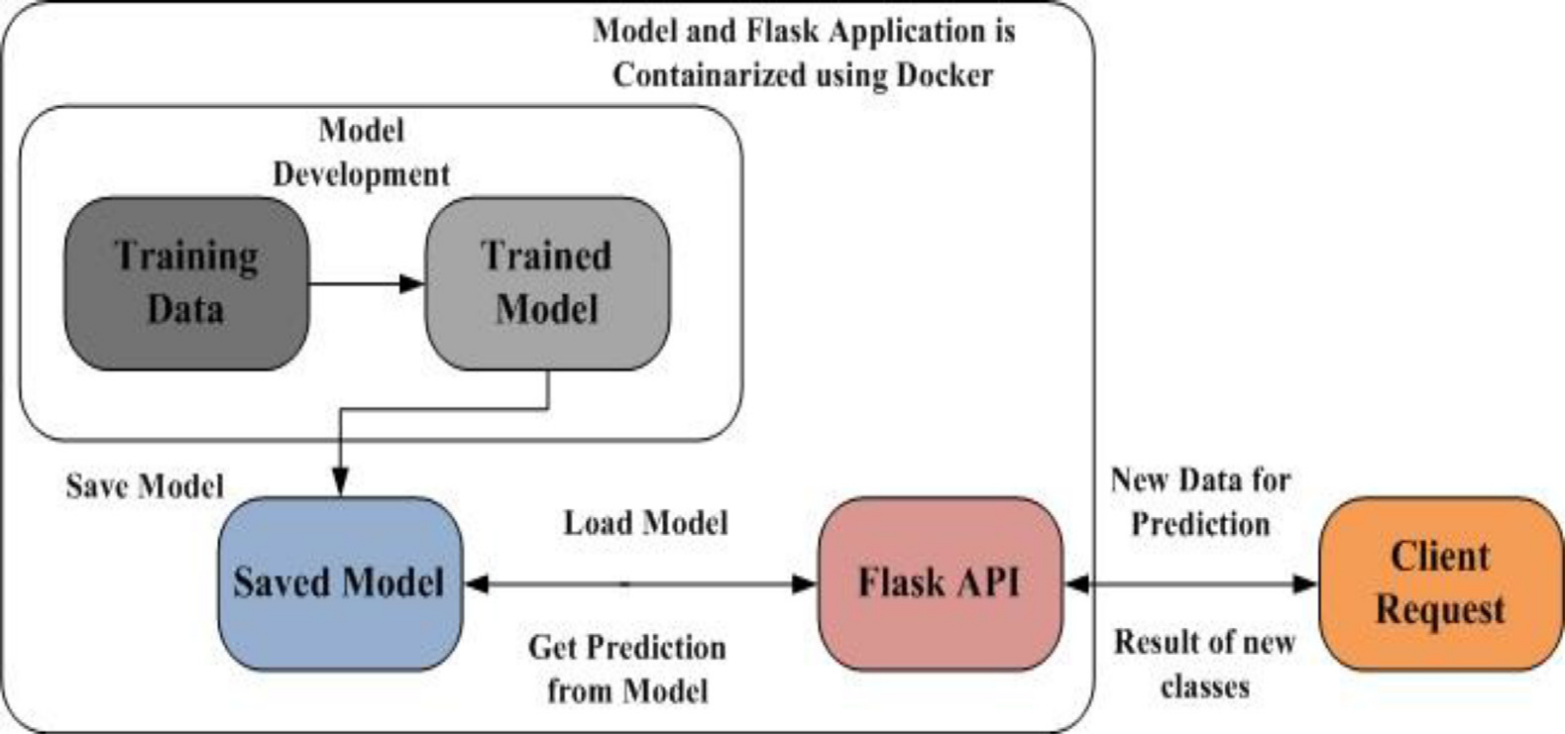
Model deployment for alert management.

Trained Model with good accuracy is saved using Serialization techniques like pickle. Flask based responsive web dashboard is designed to interact with end-users. Client will validate or test model by providing new data to flask API, which in-turn loads the saved model and passes clients data to model, get the prediction from model and passes result of new classification to end-user. In-order to have portability of this model on any platform this work wraps or packages model, Flask API and other software packages, run time libraries in to Docker image. So this Docker image is saved in Docker hub registry and can run on any Docker host irrespective of operating system and hardware and software platform. This process is depicted using Fig. 5.

As Flood Alerting System is based on Web services/API/Web Dashboard kind of design, Government Officials and Public can upload the relevant data about Temperature, Humidity, Rainfall, Reservoir Level, Vegetation Cover etc. of the relevant area and can predict the Flood level immediately with no delay or latency.

## Data collection

Quantitative data types are selected for building Flood prediction models. Literature review reveals that Rainfall, Lack of vegetation, broken dams, soil erosion, poor drainage facility, Storm Surges and Tsunamis, rivers filled with silt, Melting Snow and Ice are the major cause of Floods. Proposed work has synthesized secondary data to prove its significance in building efficient flood prediction model. Data considered for the development of model includes:

**Historical Rainfall:** Data of a particular geographical location over a period of 10 years from 2006 – 2016 archived from Kaggle Dataset [24].

**Reservoir Level**: Karnataka State Natural Disaster Monitoring Centre, an official website of Government of Karnataka State [25].

**Underground Drainage Water Level:** in the region captured by concerned public authorities - Microcontroller based embedded system is used monitor status of Drainage system. Level, Overflow and Flow sensors are used to measure status of underground drainage using Microcontrollers and ESP32/Node MCU is used to ingest Drainage data in to data lake via Wi-Fi.

**Vegetation/ Forest cover of geographical location**: Real time information regarding Land mass of geographical location and Tsunami data are fetched from satellite imagery from Earth explorer [26] and Geo-Wiki [27], a database of remote sensing repository dedicated for catalogs of satellite and aerial imagery.

**Tsunami Warning data:** Rainfall or Cyclone or Tsunami Alerts from IMD web-sites [28].

## Data analysis

The proposed work conducts data analysis on synthesized secondary data to demonstrate that critical flood causing parameters such as rainfall, reservoir level, and underground drainage water level, among others, have statistical relevance in flood outcome prediction. Following are the results of Exploratory data analysis on synthesized dataset.

### Descriptive statistics analysis of data

**Fig. 6:**
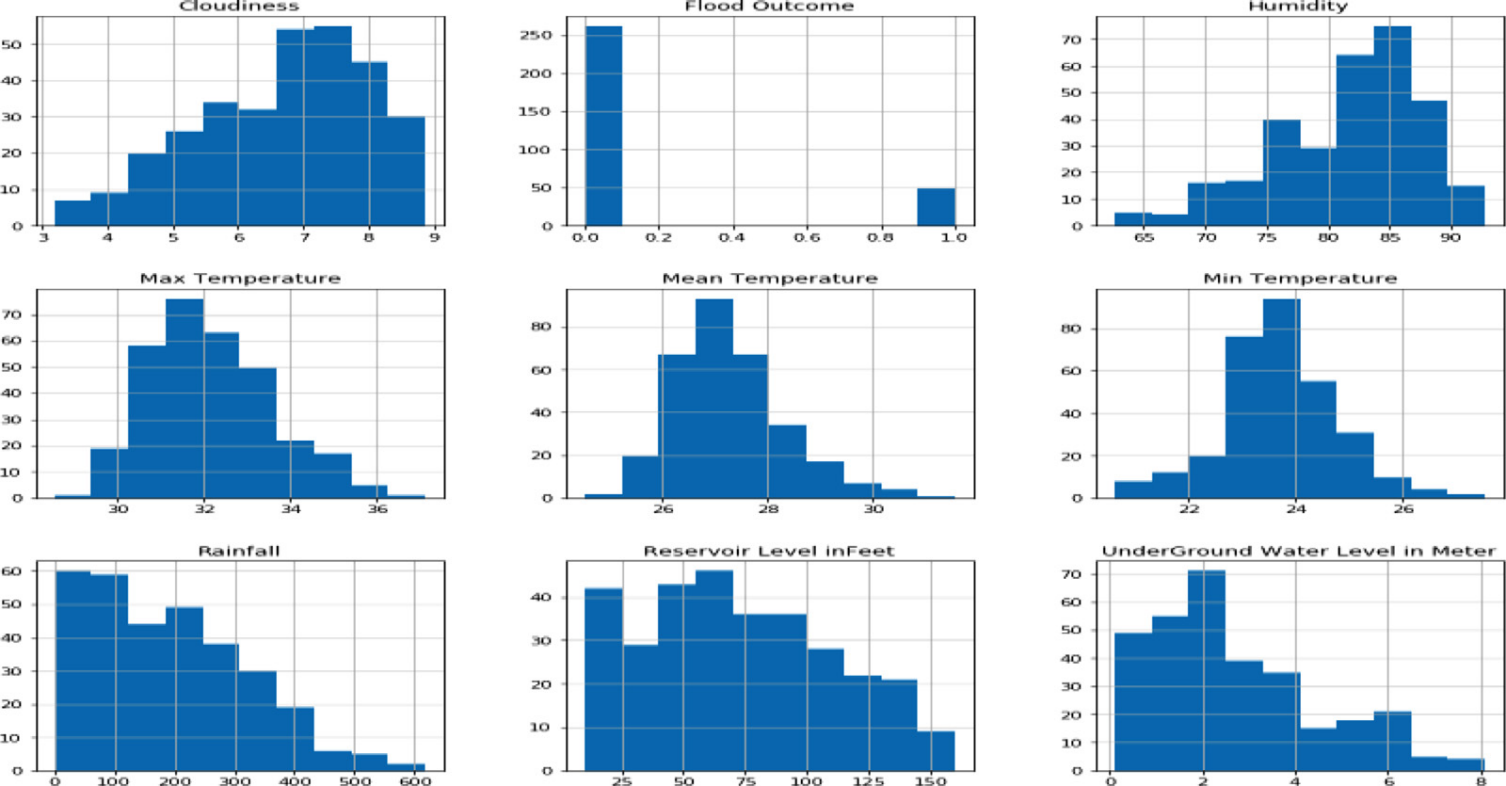
Histogram plot of data fields.

Attribute Statistics: Describes shape of any selected fields in a structured dataset. Attribute statistical information are visualized using Histogram plots (Fig. 6). Histogram plot describing attribute statistics of dataset fields.

**Fig. 7:**
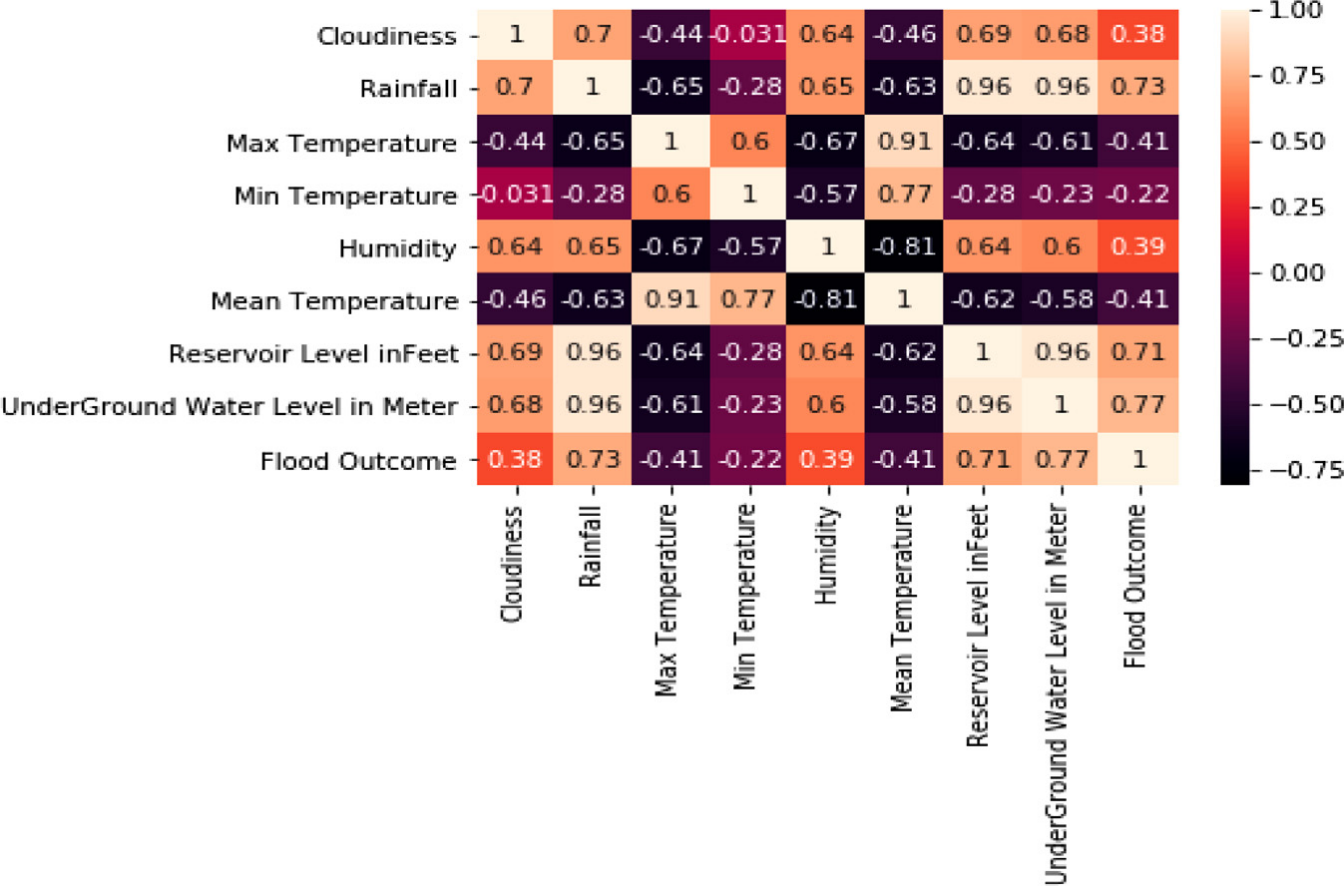
Correlation between fields of dataset.

Multi-Variate Statistics: Relationship or correlations between fields of dataset are understood using Pair-plot and Correlation Matrix. Fig. 7 is the correlation results of multi-variate statistics on dataset fields. Results of the plot show that Rainfall, Reservoir level and Underground drainage water level have high correlation of 0.70 and above on Flood outcome variable of dataset.

From the analysis study infer there exist high correlation between Rainfall, Reservoir level, Underground drainage water level on Flood outcome. Hence study infers that factors considered in dataset have statistical significance on endogenous variable i.e. Flood outcome, so this leads to design of an efficient flood prediction model in comparison to current state-of-art flood prediction models.

### Inferential statistics

1. Pairwise Pearson Correlations between Rainfall and Reservoir level
  Inference Drawn from Hypothesis-1: There is significant association between Rainfall and Reservoir Level. Results are tabulated in Table 1.

**Table 1:** Correlation between rainfall and reservoir level in feet with P-value.

Sample 1	Sample 2	N	Correlation	95% CI for ρ	P-Value
Rainfall	Reservoir Level in Feet	312	0.954	(0.942, 0.963)	0.000
2. Pearson Correlation Statistical test performed to determine the association between Rainfall and Underground Drainage level. Results are depicted in Table 2.

**Table 2:** Correlation between under ground water level in meter with P-value and rainfall.

Sample 1	Sample 2	N	Correlation	95% CI for ρ	P-Value
Rainfall	Under Ground Water Level in Mete	312	0.959	(0.948, 0.967)	0.000

Inference Drawn from Hypothesis-2: There is significant association between Rainfall and Under Ground Water Level.

3. Binary Logistic Regression: Flood Outcome versus Rainfall, Reservoir Level in Feet, Under Ground Drainage Water Level in Meter. Regression Coefficients and odds ratio for predictors of regression analysis are represented in Tables 3 and 4.

**Table 3:** Regression co-efficient table.

Term	Coef	SE Coef	Z-Value	P-Value	VIF
Constant	-135.8	47.2	-2.88	0.004	
Rainfall	0.2129	0.0781	2.73	0.006	4.57
Reservoir Level in Feet	0.786	0.341	2.31	0.021	19.42
Under Ground Water Level in Meter	-5.72	4.52	-1.27	0.206	11.09

**Table 4:** Odds ratios for continuous predictors.

	Odds Ratio	95% CI
Rainfall	1.2373	(1.0617, 1.4419)
Reservoir Level in Feet	2.1956	(1.1250, 4.2851)
Under Ground Water Level in Meter	0.0033	(0.0000, 23.0547)

Regression Equation

(1) $$P\left(1\right)=\frac{\exp\left(Y^{|}\right)}{1+\exp\left(Y^{|}\right)}$$

(2) $$Y^{|}=\;-135.8\;+\;0.2129\;Rainfall\;+\;0.786\;Reservoir\;Level\;in\;Feet\;-\;5.72\;Under\;Ground\;Drainage\;Water\;Level\;in\;Meter$$

**Odds Ratios for Continuous Predictors**

**Model Summary**

Inference drawn from Regression Test is that Rainfall, Reservoir level and Underground water level have a significance impact on endogenous variable i.e. Flood outcome as shown in Table 5.

**Table 5:** Model summary.

Deviance R-Sq	Deviance R-Sq(adj)	AIC	AICc	BIC	Area Under ROC Curve
98.92%	97.81%	10.94	11.07	25.91	1.0000

## Implications of the study

In this study Flood Prediction model is designed considering Flood causal factors like Rainfall, Reservoir Level, Underground Drainage water level. Future work would be considering other Flood causing factors like landmass cover, Vegetation, Soil erosion etc. Flood warning is implemented using Web based dashed and alerts to authorities via REST API/Web services. Future work can include warning system using Wi-Fi and Mobile notification services. Flood prediction model helps authorities in early planning for preparedness to minimize loss of asset and life.

## Conclusion

The proposed study is based on the discovery of a research gap that has to be addressed. This study is a systematic application of IoT, Big Data, and Statistical Analysis in identification, collection and analysis of important Flood causing parameters. Study analyze statistical significance of data in design of efficient flood prediction model by descriptive statistical analysis and hypothesis testing using inferential statistical analysis. Future scope to synthesize data and set-up Data Lake to ingest heterogeneous flood causing variables, design an efficient flood prediction model and finally deploy model as warning system to alert stakeholders. The findings of this study will greatly assist government officials and affected people in making decisions in the event of a severe flood, decreasing socioeconomic losses.

## Declaration of Competing Interest

Please tick the appropriate statement below (please do not delete either statement) and declare any financial interests/personal relationships which may affect your work in the box below.

The authors declare that they have no known competing financial interests or personal relationships that could have appeared to influence the work reported in this paper.

## Data Availability

No data was used for the research described in the article.
